# Comparison of the Efficacy of Budesonide Nebulizer Suspension and Budesonide Inhalation Suspension in the Treatment of Acute Exacerbation of Chronic Obstructive Pulmonary Disease

**Published:** 2018-02

**Authors:** Mohammad Emami Ardestani, Elham Klantar, Mahdi Azimian

**Affiliations:** Department of Internal Medicine, Isfahan University of Medical Sciences, Isfahan, Iran.

**Keywords:** Nebulizer, Turbuhaler, Budesonide, Chronic Obstructive Pulmonary Disease

## Abstract

**Background:**

Inhaled corticosteroids have been successfully used to improve lung function. Budesonide nebulizer suspension and Budesonide inhalation suspension are two inhaled corticosteroids used in treating chronic obstructive pulmonary disorder (COPD). We compared the efficacy of Budesonide nebulizer suspension and Budesonide inhalation suspension in the treatment of patients with acute exacerbations of COPD, to prioritize the two treatments.

**Materials and Methods:**

In our study, 90 patients were recruited and divided randomly into two groups: Budesonide nebulizer suspension (Pulmicort Nebulizer) and Budesonide inhalation suspension (Pulmicort Turbuhaler) groups. Demographic characteristics, patient clinical information, and paraclinical data including arterial blood gases (ABG) and O_2_ Saturation were recorded both at the beginning of hospitalization and on the seventh day of treatment. The collected data were analyzed through independent sample *t*-test, paired sample *t*-test, chi-square test, and linear regression using SPSS version 20.

**Results:**

Our findings revealed that there were no significant differences in O_2_SAT or ABG between the two groups at baseline or after seven days of treatment (*P* > 0.05). After seven days of treatment, mean O_2_SAT and arterial blood gases had increased significantly in each group (P < 0.001).

**Conclusion:**

This study found no significant difference between the two groups with respect to the method of treatment. Therefore, Turbuhaler can be used at home by patients so, it leads to elimination of costs and hospital stays.

## INTRODUCTION

Chronic obstructive pulmonary disease (COPD) is characterized by irreversible limitation of airflow while breathing. COPD patients usually experience episodes of deterioration that are accompanied by abnormal inflammatory reactions in the lungs to toxic gases or particles. COPD was the sixth-leading cause of death worldwide in 1990, and it is projected to be the third-leading cause of death 2020. COPD is one of the leading causes of death in developing countries, and the number deaths caused by COPD continues to increase (1–3).

One of the key goals in controlling airway inflammation is to remove obstructions of the airways and to improve ventilation as soon as possible (4). Inhaled corticosteroids are the most effective anti-inflammatory medications used in the treatment of asthma (5). Such medications reduce the number of inflammatory cells and their activities in airways, and they are useful in treating asthma of any severity at any age (5–7). Many studies have shown that using inhaled corticosteroids in the treatment of patients who repeatedly experience acute exacerbations can decrease the severity of COPD by up to 25%. There is some evidence that inhaled steroids are effective in treating acute exacerbations of COPD and lead to increases in forced expiratory volume (FEV) (8–12).

Regular use of inhalant corticosteroids can be influential in improving lung function. Therefore, inhaled corticosteroids are usually used for treating hospitalized patients to decrease the length of stay in hospital, to decrease recurrence of exacerbations, or to accelerate recovery. Pulmicort Nebulizer and Pulmicort Turbuhaler are two inhaled corticosteroids that are used to treat COPD.

The aim of the present study was to identify and compare the effects of Pulmicort Nebulizer and Pulmicort Turbuhaler in the treatment of patients with exacerbations of COPD in order to identify a preferred treatment.

## MATERIALS AND METHODS

**Figure 1.**
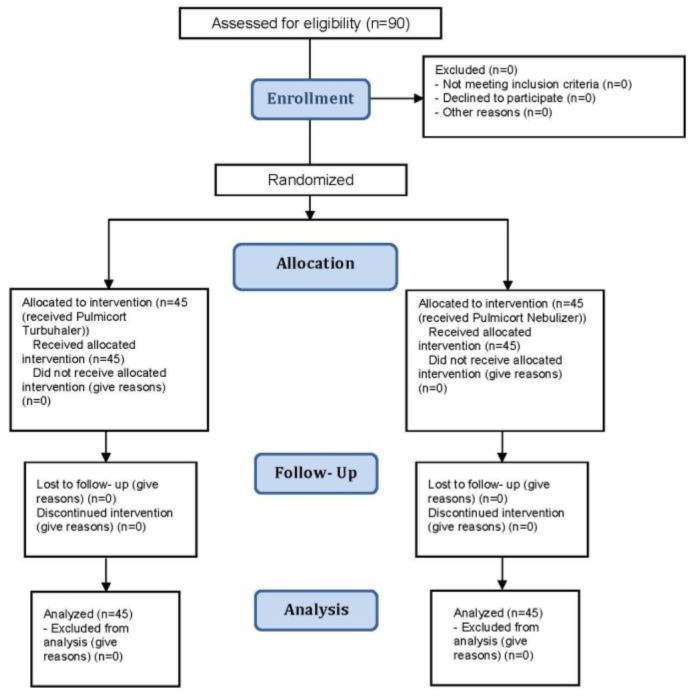
Consort flow diagram

The present study conducted among COPD patients referred to, and hospitalized in, Alzahra Hospital in Isfahan in 2015–2016. The sample size of 45 patients in each group was determined based on a confidence level of 95%, a statistical power of test of 80%, a prevalence of recovery of 0.5 in patients treated with Pulmicort, and a margin of error (d)=0.3 between these two groups. The sample consisted of patients with COPD—as determined by their clinical signs, spirometry results, chest radiography, and arterial blood gas (ABG) tests—who were admitted to the pulmonary department of Alzahra Hospital during 2015–2016. The patients were assigned to two groups using a random number table. All patients met the criteria for stages 1 through 4 COPD, according to Global Initiative for Obstructive Lung Disease (GOLD) guidelines (Figure 1).

According to GOLD guidelines defining acute exacerbations of COPD, the inclusion criteria were acute respiratory distress, increased duration and severity of coughs, increased amount and purulence of phlegm, or increased wheezing in the previous 24 hours. Those who had a history of asthma or respiratory distress before the age of 35, had no spirometric data, had taken steroids orally or through injection in the first month of disease appearance, had invasive mechanical ventilation through a tracheal tube, or had any other condition resulting in hospitalization were excluded from the study.

Approval was obtained from the Ethics Committee of Research Chancellery at Isfahan University of Medical Sciences (IR.MUI.REC.1394.4.754), and written informed consent was obtained from all patients. Participants were consecutively assigned to two groups—Pulmicort Nebulizer and Pulmicort Turbuhaler—based on simple randomization.

In addition to inhaled corticosteroids, patients in both groups received a standard treatment including a third-generation cephalosporin, two puffs of salbutamol pro re nata (PRN; as needed), three puffs of ipratropium bromide (20 mcg/puff): (Atrovent, Boehringer Ingelheim, Germany) every 6 hours, daily injection of methylprednisolone 50 mg, and nasal oxygen through a mask (O_2_SAT > 92%) at the beginning of the study.

Patients in the Pulmicort Nebulizer group received 200 μg (0.8 mL) Pulmicort Nebulizer every 12 hours through an oxygen mask, and patients in the Pulmicort Turbuhaler group inhaled one 200-μg puff of Pulmicort Turbuhaler every 12 hours. Demographic characteristics and clinical information—including age, gender, length of COPD (years), severity of dyspnea (scored from 0 to 10), amount of daily phlegm (mL), and blood tests—were recorded at the beginning of hospitalization. Paraclinical data, including ABG and O_2_SAT (determined with a pulse oximeter), was also recorded both at the beginning of hospitalization and on the seventh day of treatment. In addition, patients who were discharged from the hospital were followed up for 3 months to record the number of COPD attacks that resulted in rehospitalization.

Finally, the collected data was entered into SPSS, Version 20. Quantitative variables such as mean and standard deviation, and qualitative variables such as frequency and frequency percentage, were calculated. Fisher’s exact test or the chi-square test were used to compare qualitative variables, and the *t*-test was used to compare continuous variables between two groups. To compare O_2_SAT and ABG between the two groups, independent and paired *t*-tests were used separately and on the first and 7^th^ day after intervention. To identify factors affecting ABG variations in each group, regression analysis was used to adjust for confounders. In all analyses, we considered *P* < 0.05 to be significant.

## RESULTS

The Pulmicort Turbuhaler group (n = 45) consisted of 34 males (75.6%) and 11 females (24.4%) with a mean age of 65.96 ± 10.39. The Pulmicort Nebulizer group (n = 45) consisted of 27 males (60.0%) and 18 females (40.0%) with a mean age of 69.96 ± 11.85. Statistically, both groups were the same in terms of age and gender (*P* > 0.05). There was no significant difference between the two groups in terms of COPD stage (*P* > 0.05).

Other clinical characteristics, including severity of dyspnea, amount of daily phlegm, number of COPD attacks, and laboratory results, are presented in Table 1.

**Table 1.** Patient Baseline and clinical Characteristics

**Characteristics**		**Pulmicort Turbuhaler**	**Pulmicort Nebulizer**	**P value**
		**(n=45)**	**(n=45)**	
**Gender**	**Male**	34(75.6)	27(60)	0.114
	**Female**	11(24.4)	18(40)	
**Age, year**		65.96(10.39)	69.96(11.85)	0.092
**Current smoker**		28(62.2)	22(48.9)	0.203
**Lung disease duration, year**		5.11(3.26)	4.72(2.22)	0.056
Stages of COPD according GOLD guideline^*^	**Stage 1**	10(22.2)	9(20)	0.856
	**Stage 2**	20(44.4)	17(37.8)	
	**Stage 3**	12(26.7)	15(33.3)	
	**Stage 4**	3(6.7)	4(8.9)	
**Drug history**	**Seroflo inhaler**	24(53.3)	23(51.1)	0.833
	**Symbicort Turbuhaler**	21(46.7)	22(48.9)	
	Atrovent Inhaler^**^	45(100)	45(100)	
**severity of dyspnea(0–10)**		4.80(1.18)	5.20(1.08)	0.097
**Amount of Daily phlegm, cc**		14.11(11.74)	11(6.96)	0.130
**Number of COPD attacks**				
**Any**		4(8.9)	2(4.4)	
**1–2 times**		18(40)	17(37.8)	0.644
**>2 times**		23(51.1)	26(57.8)	
**CXR Findings**	**Normal**	37(82.2)	39(86.7)	0.561
	**Pneumonia**	8(17.8)	6(13.3)	
**HRCT Finding**	**Normal**	35(77.8)	36(80)	0.796
	**Pneumonia**	10(22.2)	9(20)	
**O_2_ Sat (with O_2_)**		91.34(3.64)	92.62(2.48)	0.054
**WBC**		8944.44(3389.53)	8442.22(2869.07)	0.450
**NEUT**		68.88(14.22)	72.30(12.85)	0.234
**Lymp**		21.19(12.79)	18.35(11.12)	0.264
**Hb**		13.42(1.67)	13.62(2.53)	0.660
**BUN**		22.64(1057)	22.75(12.67)	0.964
**Cr**		1.17(0.35)	1.09(0.21)	0.182
**Na**		141.62(3.12)	141.09(3.69)	0.461
**K**		4.87(0.89)	4.83(0.68)	0.811
**Albumin**		3.70(0.42)	3.82(0.44)	0.199
**AST**		33.49(7.19)	39.01(27.37)	0.051
**ALT**		24.84(10.62)	28.08(13.26)	0.065
**ALK**		222.98(70.37)	251.51(86.75)	0.090
**PT**		13.47(5.90)	12.85(1.89)	0.501
**PTT**		33.53(8.33)	36.27(16.56)	0.325
**INR**		1.39(0.70)	1.27(0.17)	0.238

Data shown n (%) or mean (SD)

*: Stages of COPD according GOLD guideline are: Stage 1: Very mild COPD with a FEV1 about 80 percent or more of normal, Stage 2: Moderate COPD with a FEV1 between 50 and 80 percent of normal, Stage 3: Severe emphysema with FEV1 between 30 and 50 percent of normal, Stage 4: Very severe COPD with a lower FEV1 than Stage 3, or those with Stage 3 FEV1 and low blood oxygen levels.

**: It should be noted that atrovent treatment was used for all patients.

The results showed that there was no significant difference in O_2_SAT or ABG between the two groups at the beginning of the study (*P* > 0.05). However, after 7 days of treatment, mean O_2_SAT increased significantly in both Pulmicort Turbuhaler (16.04) and Pulmicort Nebulizer groups (14.57), (*P* < 0.001). Results of ABG tests revealed that the pH level increased significantly after 7 days of treatment, while the partial pressure of carbon dioxide (pCO_2_) decreased significantly and bicarbonate (HCO_3_) increased significantly in both groups (*P* < 0.05). However, there was no significant difference between the two groups with respect to the method of treatment (Table 2).

**Table 2.** Comparison of O_2_ Sat and ABG factors on the first and seven day between the two groups

**Variables**		**Pulmicort Turbuhaler(n=45)**	**Pulmicort Nebulizer(n=45)**	P value^*^
**O_2_ Sat (without O_2_)**	**First day**	77.02(6.52)	79.44(6.96)	0.092
	**Seventh day**	93.06(3.68)	94.01(4.11)	0.251
	P value^**^	<0.001	<0.001	
pH	**First day**	7.30(0.056)	7.32(0.039)	0.052
	**Seventh day**	7.40(0.059)	7.39(0.064)	0.443
	P value^**^	<0.001	<0.001	
**Pco_2_**	**First day**	53.96(12.85)	57.87(13.07)	0.156
	**Seventh day**	46.72(9.18)	48.25(11.56)	0.540
	P value^**^	0.002	<0.001	
**Hco_3_**	**First day**	21.17(5.97)	22.07(6.63)	0.500
	**Seventh day**	23.35(4.02)	24.68(4.79)	0.157
	P value^**^	0.045	0.035	

Data shown mean (SD).

* Level of Significance in comparison between two groups,

**: Level of Significance in comparison between first and seven day in each of groups

In evaluating ABG factors, the results showed that the severity of dyspnea and the number of COPD attacks could have a significant effect on pH factors in the Pulmicort Turbuhaler group. The severity of dyspnea could also have a significant influence on both pCO2 and HCO3 factors; more acute dyspnea was associated with a smaller decrease in pCO2 and a smaller increase in HCO3 (P < 0.05). However, the severity of dyspnea had no significant effect on the levels of pCO2 or HCO3 in the Pulmicort Nebulizer group. The higher severity of dyspnea can be accompanied with decreased blood acidity (beta= −0.047, P =0.022). In some certain circumstances in which the patient condition can be intensively affected by those factors, it would be better but in common conditions and lack of cofounders it seems that turbuhaler is enough effective (Table 3).

**Table 3.** Regression analysis to identify factors affecting the changes of ABG in each of the two study groups

**Variables**	**Factors**	**Pulmicort Turbuhaler(n=45)**			**Pulmicort Nebulizer(n=45)**		

		**Beta**	**SE**	**P value**	**Beta**	**SE**	**P value**
**pH**	**Severity of dyspnea**	−0.034	0.012	0.007	−0.047	0.019	0.022
	**Number of COPD attacks**	−0.033	0.010	0.002			
**Pco_2_**	**Severity of dyspnea**	5.899	2.636	0.033			
**Hco3**	**Severity of dyspnea**	−3.111	1.477	0.044			

The factors of age, gender, severity of dyspnea, amount of Daily phlegm, number of COPD attacks and HRCT were entered in the model as independent variables. pH, Pco2, Hco3 changes: Subtraction of the seventh-day values from the first-day values

Finally, three-month follow-up showed that there were 7 cases (15.6%) in the Pulmicort Turbuhaler group and 4 cases (8.9%) in the Pulmicort Nebulizer group who were hospitalized due to repeated exacerbations of COPD (Table 4).

**Table 4.** Number of COPD attacks during the 3-month follow-up resulted in hospitalization

**Attacks COPD**	**Pulmicort Turbuhaler(n=45)**	**Pulmicort Nebulizer(n=45)**
**Any**	38(84.4)	41(91.1)
**Rehospitalization due to attacks COPD**	7(15.6)	4(8.9)

Data shown n (%)

## DISCUSSION

COPD is one of most common chronic airway disorders that is both treatable and preventable by inhibiting chronic inflammatory responses. The best choice for treatment is the use of anti-inflammatory drugs, especially corticosteroids (13). There are many ways to deliver drugs to COPD patients, two of which are the nebulizer (14) and the Turbuhaler (15). In the current study, we evaluated the efficacy of two common treatments to find the best method for reducing the duration of hospitalization, reducing costs, and providing ease of use.

We chose budesonide (200 μg twice daily for 3 months) for this study. Johnell et al. showed that COPD patients using budesonide Turbuhaler did not have any clinical complications in 36 months of follow up (16). Furthermore, investigations into high-dose inhaled corticosteroids (Pulmicort Turbuhaler, 1,600 μg per day) revealed that dyspnea was significantly ameliorated (17). On the other hand, long-term treatment with Pulmicort Turbuhaler (800 μg in the morning and 400 μg in the evening for 6 months, and 400 μg twice daily for 30 months) has not been shown to be beneficial in the treatment of declining lung function in COPD patients (18). Although a previous study found Pulmicort to be the best choice for improving peak inspiratory flow rate (PIFR) and reduced exacerbations in vitro over dry powder inhaler (19), a combination of budesonide with other drugs—such as eformoterol—in a dry powder inhaler has been shown to be more beneficial in decreasing clinical symptoms and exacerbations in COPD patients (20). The nebulize Pulmicort with oral prednisolone could be an effective alternative (21).

The present study showed dramatic improvement in O_2_SAT and ABG in both groups up to one week after treatment initiation; there was no significant difference between the two methods. We can conclude that, although treatment with Pulmicort Nebulizer is not more effective than treatment with Pulmicort Turbuhaler, it can be equally effective in improving a patient’s clinical status. In comparing recommendations from the most recent guidelines (22) with our results, the outcomes of both groups were satisfactory and neither treatment was found to be superior to the other.

Evaluation of ABG factors in each of the two treatment groups showed that the recovery of patients treated with Pulmicort Turbuhaler can be affected by the severity of dyspnea or the number of attacks. Increased severity of dyspnea resulted in a reduction in pCO_2_ and an increase in HCO_3_ levels, which consequently leads to a decrease in blood acidity. This issue could be influenced by the number of attacks experienced by a patient. On the other hand, improvement in patients treated with a nebulizer was affected by blood pH in that higher blood acid levels in patients with dyspnea reduced improvement in outcomes, but pCO_2_ and HCO_3_ levels were not significantly influenced by blood acidity.

It is possible that treatment with the Pulmicort Nebulizer was less affected by uncontrollable confounding factors compared with the Pulmicort Turbuhaler. We demonstrated that both groups are similar in this respect, but nebulized form is an invasive approach which is recommended in case of serious illness with higher severity of dyspnea. Given that in other cases turbuhaler has a same efficacy, we can benefit from a noninvasive approach instead. On the other hand, a study on the preference and ease of using the Pulmicort Turbuhaler compared with using a pressurized metered-dose inhaler showed that patients preferred the Pulmicort Turbuhaler since it requires less time to master correct use (23). In addition, a study of local side effects during 4 years of treatment with inhaled corticosteroids revealed a lower incidence of complications (such as hoarseness and oropharyngeal candidiasis) using Turbuhaler than nebulizer (24).

At the end of the study, 15.6% of Pulmicort Turbuhaler users and 8.95% of Pulmicort Nebulizer users reported having attacks that led to hospitalization; the difference between groups was not statistically significant.

## CONCLUSION

This study demonstrated that the Pulmicort Nebulizer and Pulmicort Turbuhaler approaches were equally efficacious in improvements of O2SAT, ABG factors, and hospital stays.
